# Carbon Nanohorn-Based Electrocatalysts for Energy Conversion

**DOI:** 10.3390/nano10071407

**Published:** 2020-07-19

**Authors:** Antonia Kagkoura, Nikos Tagmatarchis

**Affiliations:** Theoretical and Physical Chemistry Institute, National Hellenic Research Foundation, 48 Vassileos Constantinou Avenue, 11635 Athens, Greece; akagkoura@eie.gr

**Keywords:** carbon nanohorns, electrocatalysis, fuel cell, oxygen reduction, methanol oxidation, water splitting, hydrogen evolution, oxygen evolution, energy applications

## Abstract

In the context of even more growing energy demands, the investigation of alternative environmentally friendly solutions, like fuel cells, is essential. Given their outstanding properties, carbon nanohorns (CNHs) have come forth as promising electrocatalysts within the nanocarbon family. Carbon nanohorns are conical nanostructures made of sp^2^ carbon sheets that form aggregated superstructures during their synthesis. They require no metal catalyst during their preparation and they are inexpensively produced in industrial quantities, affording a favorable candidate for electrocatalytic reactions. The aim of this article is to provide a comprehensive overview regarding CNHs in the field of electrocatalysis and especially, in oxygen reduction, methanol oxidation, and hydrogen evolution, as well as oxygen evolution from water splitting, underlining the progress made so far, and pointing out the areas where significant improvement can be achieved.

## 1. Introduction

**Figure 1:**
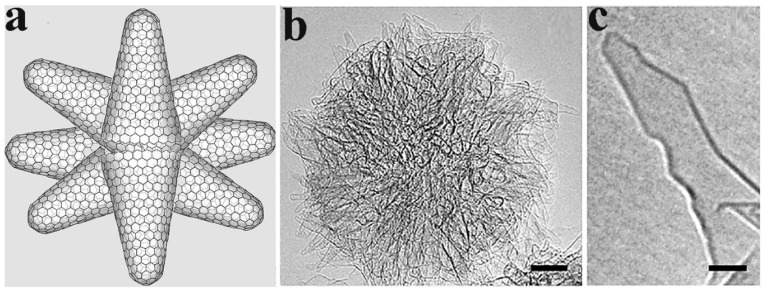
(**a**) Schematic illustration and (**b**) HRTEM image of carbon nanohorn “dahlia” aggregate (scale bar 10 nm). (**c**) HRTEM image of an individual carbon nanohorn (scale bar 2 nm). Reproduced with permission from [6]. Copyright © American Chemical Society, 2005

Carbon nanohorns (CNHs), discovered in 1998, are interesting nanomaterials due to their special morphology and physicochemical properties [1,2]. In general, CNHs consist of sp^2^ hybridized carbon atoms that form conical structures of 2–5 nm in diameter and 40–50 nm in length. The structure of CNHs contains single-graphene tubules, with highly strained conical ends, aggregated in larger spherical angled superstructures of approximately 100 nm in diameter (Figure 1), resembling the dahlia flower. In those superstructures, CNHs are tightly bound through van der Waals forces [3], and the aggregated superstructure gives additional benefits to CNHs due to the presence of external micropores and internal mesopores. On the other hand, aggregated CNHs are hard to separate and individualize. Nevertheless, the aggregated superstructure was successfully broken down by the reductive dissolution of raw CNHs in potassium naphthalenide [4]. More recently, in a simpler and less demanding approach, individualization of CNHs was achieved by treatment with chlorosulfonic acid, where the isolated tubules were found to be p-doped [5].

Markedly, CNHs’ morphology differs from that of carbon nanotubes (CNTs) due to their (a) conical tips consisting of 5-membered rings, (b) significantly smaller size, and (c) 3D spherical aggregation. The aforementioned alterations in shape and size of CNHs vs. CNTs affect their properties and, therefore, their applications. Specifically, CNHs exhibit great specific surface area, high porosity, good electrical conductivity, high thermal and chemical stability, and therefore, present exceptional catalytic properties. Another essential advantage of CNHs over CNTs is that they are produced at room temperature in industrial quantities by either CO_2_ laser ablation of pure graphite or pulsed arc discharge of carbon rods. Most importantly, in the production of CNHs, toxic metal catalysts requiring subsequent strong acidic treatment to eradicate them are absent.

**Figure 2:**
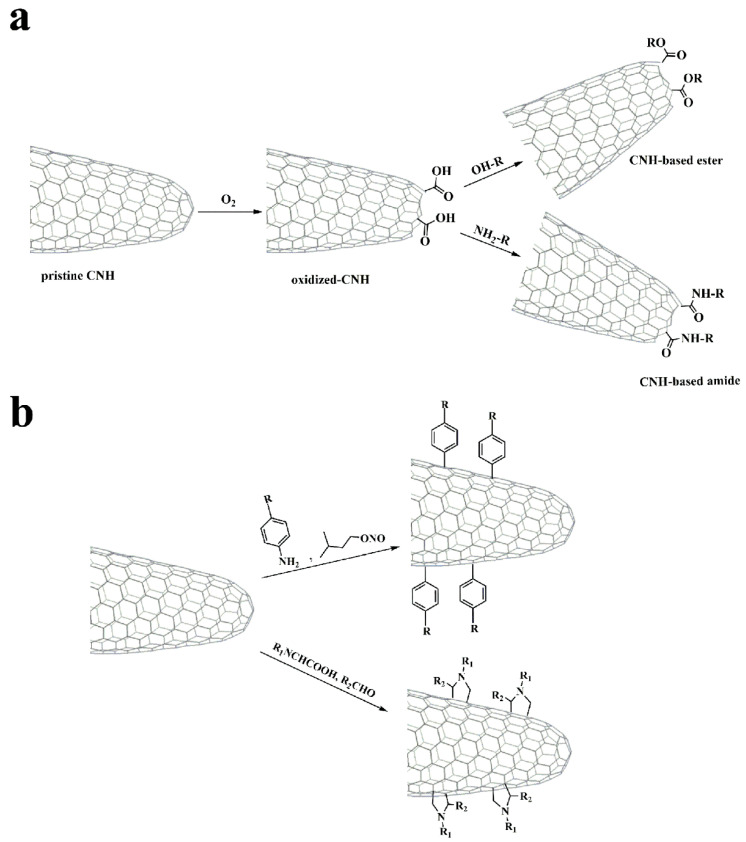
(**a**) Oxidation of CNHs and further post-functionalization of oxidized CNHs. (**b**) Functionalization of CNHs with in situ-generated aryl diazonium salts and via 1,3-dipolar cycloaddition reaction of in situ-generated azomethine ylides.

**Figure 3:**
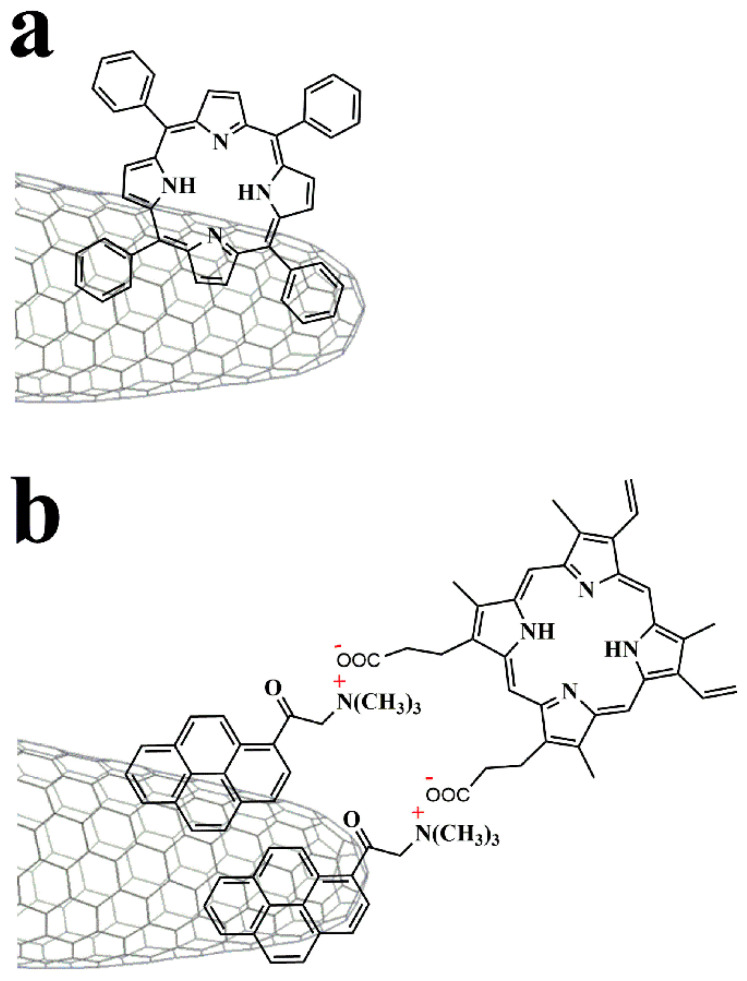
Non-covalent functionalization of CNHs with (**a**) porphyrin through by π–π stacking interactions, and (**b**) ammonium-pyrene/carboxylate-porphyrin through π–π stacking and electrostatic interactions.

Like CNTs, carbon nanohorns lack solubility, which makes manipulation and subsequent exploitation difficult. In this context, functionalization is an effective way to increase the solubility of CNHs and allow further modification with other species. Particularly oxidation of the conical tips, where most of defects are located, opens the conical tip of CNHs, further increases their surface area, and also gives direct access to the interior space. The covalent modification approaches involve either the introduction of carboxylic units suitable for further post-functionalization at the conical tips of CNHs upon oxidation [3,7,8,9,10,11,12,13,14] or the direct attachment on the outer skeleton of CNHs of organic addends (Figure 2), mostly via cycloaddition reactions [15,16,17,18,19,20,21,22,23,24,25,26,27,28,29,30,31,32,33,34]. Meanwhile, plenty of strategies have been also developed for the non-covalent functionalization of CNHs (Figure 3), mainly including electrostatic [35,36,37] and supramolecular π–π interactions [38,39,40], keeping unperturbed their electronic properties.

The functionalization of CNHs with organic species has led to the realization of hybrid materials for energy conversion [41] and storage [42], biosensing [43], gas storage [44], and optoelectronic devices [45]. Moreover, the chemical defects and crystal edges of CNHs confer good electrocatalytic properties and render them suitable candidates in electrocatalysis [41]. Specifically, CNHs have been engaged principally in vital electrocatalytic reactions involved in fuel cell technologies and/or production of renewable fuels, mostly as supports for the realization of hybrid materials, contributing to higher performances and better stability and durability. This review highlights the advances made with CNH-based materials in electrocatalysis towards oxygen reduction, methanol oxidation, and hydrogen evolution as well as oxygen evolution from water splitting.

## 2. CNHs in Electrocatalysis

### 2.1. Oxygen Reduction

Electrocatalysis provides a sustainable and efficient energy process, with electrolysis and fuel cells being auspicious application-platforms, for energy conversion. Fuel cells are electrochemical-based devices that are considered to be the most promising power sources for stationary and portable electronic devices as well as transportation. Analogous to batteries, fuel cells convert chemical energy of a fuel directly into electric energy. Most proton exchange fuel cells (PEMFCs) are powered by hydrogen, which can be fed to the fuel cell directly or can be generated within the system by reforming hydrogen-rich fuels. Unlike batteries, fuel cells do not need recharging as they require continuous sources of fuel (and oxygen) in order to keep the process going. Conversely, when fuel cells use hydrogen as fuel, only electricity, water, and heat are produced. Their high efficiency, no environmental pollution, and unlimited reactant sources boost them on top of other thermal engines. Notably, PEMFCs have received great attention in recent years for potential use in vehicles, portable electronics, and into combined heat and power systems due to their simplicity, high power density, quick start-up, and low working temperature. Fuel cells are being developed and tested in trucks, buses, boats, motorcycles, and bicycles, among other kinds of vehicles, and are expected to be widely commercially used as a solution in global energy problems [46].

The main reactions involved in PEMFCs are fuel oxidation at the anode and oxygen reduction at the cathode. At the anode, hydrogen is oxidized, and electrons and protons are produced (H_2_ → 2H^+^ + 2e^−^) and transferred to the cathode through an external circuit and the proton exchange membrane, respectively. At the cathode, oxygen reacts with protons and electrons and is reduced to produce water (1/2O_2_ + 2H^+^ + 2e_−_ → H_2_O). Both anode and cathode electrodes consist of platinum (Pt) to promote the hydrogen oxidation reaction (HOR) and oxygen reduction reaction (ORR). The HOR occurs extremely fast and requires a low Pt loading, typically less than of 0.05 mg/cm^2^ [47]. However, at the cathode, on the other hand, ORR is characterized by sluggish kinetics and much more Pt loading is required, of about ~0.4 mg/cm^2^, in order to achieve a good fuel cell performance [48]. It is, therefore, quite clear that the cathodic reaction needs to be optimized. Although Pt is the most effective electrocatalyst at the moment, its high cost and limited durability and stability have made the exploration of alternative electrocatalysts essential in order to limit or even fully replace Pt-based materials.

The ORR can occur through two possible pathways in aqueous media. One involves direct water production via a four-electron reduction route according to the following equation:

O_2_ + 4H^+^ + 4e^−^ → 2H_2_O (1)

The other is a two-electron reduction route, which involves the formation of hydrogen peroxide (H_2_O_2_) as an intermediate according to the following equations:

O_2_ + 2H^+^ + 2e^−^ → H_2_O_2_ (2)

H_2_O_2_ + 2H^+^ + 2e^−^ → 2H_2_O (3)

The most efficient and preferable reaction for the fuel cell technology is the four-electron pathway, while the formation of hydrogen peroxide is considered to be undesirable, as it leads to low efficiency. On the other hand, hydrogen peroxide is a versatile chemical with high commercial value, with applications in the pulp-and-paper, textile, synthetic chemical, and waste-water-treatment industries. Thus, recent trends in the electrochemical reduction of O_2_ require hydrogen peroxide as the desirable product, since selective synthesis of hydrogen peroxide through ORR provides a cheap, ecological, and safe way for its production [49].

Without a doubt, the investigation and exploration of novel, less expensive, and abundant electrocatalysts for the ORR is of paramount importance. Recent research has focused on highly active catalysts driving the 4e^−^ ORR, including Pt alloys [48], core-shell nanostructures [50], transition metal oxides [51], and chalcogenides [52], and carbon-based non-noble catalysts [53,54]. Carbon-based materials, such as graphene, doped graphene, and CNTs have been studied as conductive supports in ORR due to their excellent conductivity and high surface area [52,53,54,55]. Although CNTs have been tested in electrocatalysis [53], they lack certain essential characteristics for an ideal electrocatalyst, such as low preparation cost, high porosity, and high surface area. In an attempt to overcome these limitations, CNHs were used as starting material to prepare CNT-encapsulated with iron oxide nanoparticles (Fe-CNH/CNT), by annealing a mixture of CNHs, melamine, and iron salt [56]. In that way, the as-prepared nanotubes featured a tubular morphology with loose graphene layers, while they maintained the high surface area of CNHs, which was indeed the highest reported for metal encapsulated nanotubes (750 m^2^/g). The extremely stable electrocatalyst was tested towards ORR and showed lower overpotentials of 0.16 and 0.10 V vs. RHE in alkaline and acidic solutions, respectively, than reference electrocatalyst Pt/C. The Fe-CNT was also tested as cathode catalyst in PEMFCs with maximum power density of 200 mW/cm^2^, showing the prospect of this type of encapsulating carbon morphologies as Pt-free catalysts for fuel cells [56].

There is no question that doping nanocarbon materials with heteroatoms can increase their catalytic activity and selectivity towards specific reactions. Particularly, the incorporation of heteroatoms such as N, B, S, and P within the graphene skeleton induces changes in the electron density distribution, due to the electronegativity difference of the foreign element with carbon atoms, that effectively tunes the electronic states of the material [57]. This effect, along with charge density, results in improvement in the electrical conductivity of the material. Heteroatom-doped carbon-based nanomaterials have been ascribed as promising cathodes for fuel cells. Nevertheless, their catalytic ability towards ORR is still insufficient compared to Pt-based catalysts and specifically, in terms of the onset potential. The creation of adequate active sites on the electrocatalysts is imperative in order to reduce the required energy for the reduction of O_2_ and achieve high-density chemisorption of dioxygen. It is widely accepted that the edges of carbon nanostructures are more active towards doping. Therefore, incorporation of more edges and/or improvement of the porosity of the surface are two effective ways to modulate the active sites [58]. Much effort has been given into enhancing the surface area of carbon nanomaterials, including hydrothermal methods, hard- and soft-template assisted syntheses, and annealing high surface area materials, such as metal–organic frameworks (MOFs) [59,60]. However, their electronic conductivity can be affected by the synthetic procedure [61]; thereby, it is extremely important to adopt preparation routes that can ensure electrocatalysts with high surface area that maintain their electronic conductivity. Normally, CNHs possess a surface area of around 300–400 m^2^/g, while it was observed that their surface area decreases by increasing treatment temperature [62]. Furthermore, when the surface of porous carbon nanomaterials increases, the electrical conductivity is reduced, implying strong relations between electrical conductivity and porosity, both of them being needful for materials aiming at energy-device applications. Modification of CNHs can further enhance their surface from 350 to 1700 m^2^/g [62]. A simple method for tuning CNHs’ surface area is oxidation [63,64,65]. In this manner, oxidation was used to open up the tips of CNHs and achieve high electrochemical surface area [65]. The oxidized CNHs were used as support for the immobilization of Pt nanoparticles and employed as anode electrocatalysts in PEMFC, with an overpotential of ~0.63 V [66].

Heteroatom doping is definitely an efficient strategy to enhance conductivity as it favorably modulates the electron density of the carbon nanomaterial [67]. Apart from that, in situ doping of heteroatoms can increase the number of defects of CNHs without using metal catalysts [57]. In this scenario, nitrogen-doped CNHs (N-CNHs) were prepared by thermally treating hydrogen peroxide functionalized CNHs with urea in order to obtain better ORR activity [68]. The so-produced electrocatalyst possessed high surface area of >1800 m^2^/g with well-defined microporosity and electrical conductivity. More interestingly, the CNH-based electrocatalyst showed enhanced ORR activity compared to intact CNHs, originating from the pyridinic form of the doped nitrogen. N-doped CNHs showed higher durability and methanol fuel tolerance compared to Pt/C, whereas they were used as a cathode catalyst in fuel cells and provided a maximum power density of 30 mW/cm^2^. In seeking of increasing the N-doping efficiency, CNHs with controlled defects on the conical edges and sidewalls were synthesized under mild oxidation and afterwards, were treated with nitrogen plasma [69]. The N-doped CNHs demonstrated significantly improved electrocatalytic activity for ORR compared to intact CNHs, resulting from the enriched edges with pyridinic-N atoms and the good electrical conductivity and excellent mass transport guaranteed from the inherent spherical three-dimensional quality of CNHs. In this context, the influence of Fe concentration was evaluated in the preparation of sulfur-doped CNHs regarding their morphology, chemical, and electrochemical properties [70]. The modified chemical vapor deposition method with ferrocene gave the most well-defined tubular nanostructures, with conical and cone-like shapes at lower Fe amounts. Sulfur-doped CNHs exhibited fair electrocatalytic activity against ORR but still far from the commercial Pt/C. Moreover, dual-doped carbon nanomaterials can further enhance the catalytic activity by disrupting the electroneutrality of graphitic π-system due to the synergistic effect between the doped atoms [71,72]. For instance, nitrogen- and boron-doped (N-B-doped) graphene exhibited enhanced ORR compared to that owed to N-doped graphene, justified by the high density of catalytic sites and the synergistic effect between N and B atoms [73,74]. In a similar manner, co-doped vertically aligned carbon nanotubes with N and P showed enhanced ORR activity, which was mainly attributed to the morphological modification, surface area expansion induced by the doping, and the increased doping concentration [75].

Co-doped nanocarbon materials often involve tedious and expensive preparation techniques that render their mass production unrealistic. On top of that, the presence of unnecessary by-products and difficult removable metal impurities make the identification of the nature of proposed synergistic effects hard to tell. Meanwhile, the determination of the dopant source may play a crucial role in the electrocatalytic performance of the catalyst. As a proof of concept, nitrogen-boron and nitrogen-phosphorous co-doped CNHs (N-B-CNH and N-P-CNH, respectively) were prepared by using two different N-sources for each co-doped material, namely nitrogen and melamine, and boron carbide and triphenylphosphine as B and P sources by the one-step arc discharge method [76]. It was found that the co-doping of N and P synthesized by nitrogen resulted in higher N-amounts in CNHs, causing high catalytic activity towards ORR. On the other hand, N-B dual-doping of CNHs by nitrogen favored the formation of hexagonal boron nitride, which is inert in ORR, resulting in reduced electrocatalytic activity. However, this was not noticed during the incorporation of N-B atoms in CNHs with melamine as N-source, where the synergetic effect among N-C-B due to polarization of adjacent atoms helped in marking the highest electrocatalytic activity overall [76].

Undoubtedly, increasing the accessibility of available active sites is critical for the enhancement in ORR performance. It is proven that the micropores are regarded as the hosts of the majority of active sites and they are consequential for increasing the number of active sites [77]. Partially unzipped CNTs, possessing active centers with improved characteristics to reduce dioxygen, were employed [78]. Taking into account their high porosity and surface area, CNHs were used in an electrospinning process to increase the specific surface area and pore volume of carbon fibers [79]. Markedly, CNHs increased the electrical conductivity and also helped create mesopores and macropores, which are extremely effective in mass transfer processes [80]. After doping with Fe and N, a series of dual-doped carbon fibers were prepared and tested in ORR. Actually, optimal catalyst showed the best electrocatalytic activity for the reduction in O_2_ recorded so far, with a half-wave potential value of 60 mV higher than that of the commercial Pt/C, in alkaline solution. Moreover, the electrocatalyst was tested as a cathode catalyst in PEMFC and anion exchange membrane fuel cells (AEMFCs), with high power densities of 250 and 125 mW/cm^2^, respectively, demonstrating their use in real applications [79].

In seeking to prepare effective cathode electrocatalysts for ORR, diverse synthetic strategies involving various architectures on carbon supports were explored, engaging Pt bimetallic [81] or multi-metallic nanoparticles on grapheme [82], core-shell structures [50,54,83], etc. The electrical conductivity and stability of the decorated metallic nanoparticles could be effectively improved through hybridization with carbon nanostructures [50,54,84], while it could also address the lack of stability that metal catalysts face during the electrolysis process due to dissolution or agglomeration issues [85]. However, the smoothness of the carbon nanomaterial makes the interaction between the active site and the carrier very weak. In many cases, defects are held responsible for many electrochemical activities ascribed to graphitic materials. Indeed, the introduction of heteroatoms can induce defects and irregular structures of graphene. In this matter, the defected nature of CNHs due to their nanoscale pores and dahlia flower-like morphology could be more favorable for the dispersion of the nanoparticles and prevent their agglomeration compared to graphene, and thus, further promote ORR. Aiming at exploring this claim, Pd nanoparticles were uniformly loaded on N and B dual-doped CNHs, in the form of the hybrid Pd-N-B-CNHs, by a one-step reduction method and their ORR activities were studied in alkaline media [86]. Electrochemical studies showed that Pd-N-B-CNHs exhibited similar onset potential to that of the commercial Pt/C and a more positive half-wave potential by 0.167 V vs. SCE. The large pore size distribution, the presence of numerous pyrrolic-N and charged B^+^ sites, the defect carbon nanostructure, and the synergetic effect between metal and N-B-CNHs facilitated superior activity towards ORR. Additionally, the electrocatalyst showed long-term stability and resistance to methanol, underscoring the high potentiality of Pd-N-B-CNHs for fuel cells [86]. However, in some cases, the defected nature of CNHs has not worked for their benefit. Particularly, the corrosion resistance of CNH-based electrocatalysts was evaluated in strong conditions [87]. In this frame, CNHs presented a three times faster corrosion rate than that of carbon black (CB) and commercial Pt electrode. This observation is contradictory to the studies that want CNTs [88,89] to be more tolerant compared to CB due to their higher graphitic structure [90]. One would expect that CNHs will present a better oxidation profile than highly amorphous CB, however, their numerous defected sites are most likely prone to electro-oxidation. The aforementioned facts stress the importance of a good overall electrocatalyst for applying in real fuel cells.

Furthermore, most metastable nanostructures of bimetallic or multi-metallic Pt-based electrocatalysts usually use synthetic procedures concerning complicated methods such as electrochemical etching or deposition processes, which limit the application of such catalysts at a large scale [91]. In addition, when these nanostructures involve noble metals, including Pt, Pd, and Au, purification becomes more complicated than that of monometallic catalysts. Consequently, the development of supported monometallic-Pt nanoparticle catalysts with high durability and electrocatalytic activity for ORR is of high significance for promoting the utilization of PEMFCs. As a matter of fact, Pt-loaded CNH-based catalysts have shown high performance in polymer electrolyte fuel cells [92,93,94]. Additionally, Pt nanoparticles supported on N-doped CNHs (Pt/N-CNHs) exhibited enhanced durability and catalytic activity for ORR compared to commercially available Pt/C catalysts in an acidic environment [95]. Specifically, Pt/N-CNHs showed 1.6 times higher activity in terms of onset potential than the commercial Pt/C, while its catalytic activity was higher by 75% overall.

Transition metal chalcogenides (TMCs) is another category of electrocatalysts that has received attention due to their multifunctional behavior and ease of synthesis [96]. TMCs are highly tolerant towards fuels typically used in fuel cell anodes, such as methanol and formic acid. Transition metal chalcogenides (e.g., selenides and sulfides of Co, Mn, Ni, Ru, and Fe) are considered possible alternatives to Pt, since they perform well towards various electrode reactions such as oxygen reduction, oxygen evolution, and hydrogen evolution [96,97]. For example, Ru modified with Se electrocatalysts have showcased superior activity against ORR than pure metals [98] and have been combined with CB with very close activity to Pt/C [87]. Among them, CoSe_2_ and CoS_2_ have shown the most promising activities against ORR [99,100], but still not as good as of Pt-based catalysts in both acidic and alkaline conditions. This is mostly due to the instability of the selenide and sulfide at higher positive potentials [101]. In order to overcome this obstacle, CoSe_2_ nanoparticles decorated on nitrogen-doped CNHs (CoSe_2_/N-CNHs) were tested against ORR and compared to CoSe_2_ nanoparticles supported on carbon. The CoSe_2_/N-CNHs hybrid showed lower onset overpotential by 50 mV compared to carbon supported CoSe_2_. More interestingly, CoSe_2_/N-CNHs showed a half-wave potential value comparable to that of Pt/C, underlying the increase in the density of active reaction centers of CoSe_2_ due to the presence of N-CNHs as well as the synergetic effect between those two [102]. In addition, CNHs were also employed as a support for a bimetallic metal–organic framework featuring different Zn:Co molar ratios [103]. The 3D conductive network of CNHs led to an ORR performance comparable to that of Pt/C as well as excellent durability and methanol tolerance. It is also worth mentioning that the optimum electrocatalyst was used in real Zn-air battery test and made a higher peak power density (185 mW/cm^2^) than that of commercial Pt/C catalyst (160 mW/cm^2^) [103].

Fe and Co are considered promising non-precious metal counterparts for heteroatom doping, since their mutual coordination and synergetic effect with commonly used dopants (e.g., N, S, B) have proven highly effective for ORR electrocatalysis [104,105]. Their activity can be enhanced by tuning the surface area and microporosity to increase the metal-nitrogen coordinated sites. Particular emphasis has been given in using carbon nanostructures to activate the reaction centers [106], however, it is a challenge to remain intact the physicochemical properties of the carbon matrix. In this regard, the unconventional morphology of CNHs fits perfect the abovementioned criteria. Specifically, oxidized CNHs were simultaneously doped with Fe and N at 900 °C [107]. The as-prepared electrocatalyst exhibited 40% greater activity than the commercial Pt/C. Specifically, it showed more negative onset potential and half-wave potential by 20 and 30 mV, respectively, against ORR compared to Pt/C. The high electrocatalytic activity was attributed to the synergetic effect of the coordinated N atoms at the edges of the micropores of CNHs with Fe. It is worth mentioning that the ORR activity of the tested electrocatalyst was improved after 1000 cycles, while it was used as a cathode in fuel cells with maximum power density of 35 mW/cm^2^ under alkaline conditions [107].

Moreover, graphitic carbon nitride (g-C_3_N_4_), a relatively new type of carbon-based material that possesses a graphene like sp^2^ bond structure, has attracted considerable attention as a non-precious metal catalyst for ORR due to its abundant nitrogen dopants and defects. Doping of g-C_3_N_4_ with transition metals is an efficient way to improve their catalytic activity, since interactions between different transition metals and heteroatom structures do control the inherent nature of the active sites. A way to overcome the poor electroconductivity and the limited surface area that g-C_3_N_4_ face is the introduction of CNHs. Within this scope, Co-doped g-C_3_N_4_ was combined with CNHs and evaluated as electrocatalysts for ORR (Co-g-C_3_N_4_/CNHs) [108]. The CNHs’ dahlia flower morphology with high surface area, defects, and porosity aided the formation of high Co-N active sites. Thus, Co-g-C_3_N_4_/CNHs showed improved catalytic activity compared to non-doped g-C_3_N_4_/CNHs and intact CNHs, emphasizing the need for further investigating of the role of Co in these hybrid nanostructures [108].

Hydrogen peroxide is produced mainly via the anthraquinone process, which is an energy-consuming procedure depending on palladium. Because of that, there is tremendous interest in seeking alternative synthetic methods of low cost and energy. Without any doubt, electrocatalytic processes for the reduction in molecular O_2_ are quite tempting, especially those that rely on metal-free based electrocatalysts. Carbon supports and N-doped porous carbon have previously been found to be active for H_2_O_2_ production through O_2_ reduction [109,110]. The ability to tune electrocatalysts’ selectivity toward the two- or four-electron pathway is of high concern, since promotion of the ORR often only happens at high overpotentials, where the production of H_2_O is favored. In this route, N-doped graphitized CNHs (g-N-CNHs) were prepared and tested as catalysts for the ORR to produce H_2_O_2_ by combining the properties of CNHs and a unique method of N-doping [111]. Briefly, N-doped CNHs were prepared via coating and annealing of polydopamine; a process that restricted them to nanoscale. The CNH-based electrocatalyst showed excellent activity and selectivity for ORR to H_2_O_2_ at low overpotentials, outperforming current metal-based electrocatalysts. Efficient control of crucial parameters like high surface area and porosity as well as the optimal distribution of pyridinic-N and pyrrolic-N due to the polydopamine dopant led to the superior activity of the modified CNHs [111].

Table 1 summarizes the electrocatalytic properties, characteristics, and performance of CNH-based materials towards ORR.

### 2.2. Methanol Oxidation

Direct methanol fuel cells (DMFCs) are a relatively new entry into the family of fuel cells technology and are considered a subcategory of PMFCs because they use a polymer membrane as an electrolyte. DMFCs were first invented and developed in the 1990s to tackle the problem of hydrogen storage and to eliminate the need of a reformer that converts fuel to hydrogen. In DMFCs, pure methanol is used as fuel and reacts directly at the anode. However, in addition to platinum, other catalysts like Ru must be added to break the methanol bond in the anodic reaction. Methanol offers several advantages as a fuel, namely, it is cheap and has low energy power density per mass unit (20 MJ/kg), while it can be easily transported, handled, and stored. This means methanol can be supplied to the fuel cell unit from a liquid refillable reservoir, which can be kept topped up, or in cartridges which can be quickly changed out when spent. DMFCs operate in temperatures between 60 and 130 °C and are mostly used in applications with modest power requirements, such as mobile electronic devices or chargers and portable power packs. Their usage as power units in materials handling vehicles even sees commercial traction in replacing the battery power in forklift trucks. However, DMFCs are held back due to two major drawbacks: (i) the poor oxidation kinetics of the fuel, and (ii) the diffusion of methanol into the cathode. When the proton exchange membrane becomes permeable to the fuel, methanol molecules can diffuse through the membrane and are directly oxidized by oxygen on the positive electrode. This can cause a mixed potential and reduce the cathode potential and consequently, the overall performance of the cell, which eventually raises the cost of the electrode Additionally, it forces the use of dilute methanol solutions at the anode (typically 0.5–1.0 M), demanding larger quantities of water, making the size and complexity of the system bigger, thus, impractical for portable devices. These problems can be addressed by using membranes with lower permeability towards methanol and by using efficient cathode catalyst materials for oxygen reduction, which simultaneously show high tolerance toward methanol chemisorption. Several strategies have been developed to take advantage of carbon supports as anode catalysts [112]. The unique structure of CNHs is advantageous to support nanoparticles due to the thousand nanospaces between the cones of the aggregates [113]. Actually, the use of CNHs as supports to Pt and PtRu nanoparticles in DMFCs and PEMFCs has shown higher catalytic activities of 60% compared to carbon black [114,115,116]. Oxidized CNHs were used to achieve better dispensability of Pt nanoparticles and they were used as cathodes in DMFCs [117]. Markedly, CNHs-based electrocatalysts reached a power density of 76 mW/cm^2^ operating at 40 °C. In another work, different assistant agents were studied for the preparation of Pt nanoparticles supported on CNHs [118]. Results showed that ionic liquids improved the dispersibility and size uniformity of Pt nanoparticles than 4,4-bipyridine. The same went for oxidized CNHs compared to intact CNHs, while the electrocatalytic activity of Pt on oxidized CNHs for methanol oxidation was higher compared to that of Pt on CNHs [118]. However, Pt nanoparticles stabilized on oxidized CNHs exhibited poor durability. Apart from this, a novel approach was developed for the preparation of Pt nanoparticles on CNHs supports (Pt/CNHs) [119]. Most synthetic procedures require the introduction of defects and/or oxygenated groups to CNHs for the effective deposition of Pt nanoparticles, which may end up damaging their structure and result in poor properties. Contrarily, in the discussed work, formic acid was employed as a reducing agent for the preparation and immobilization of Pt nanoparticles on pristine CNHs. Moreover, Pt/CNHs exhibited higher catalytic activity and better long-term stability for the optimal amount of Pt on CNHs, for both methanol and formic acid oxidation reactions than commercial Pt/C. In the same concept, an anode catalyst consisting of Pt/Ru nanoclusters supported on N-doped CNHs (PtRu/N-CNHs) was evaluated for the methanol oxidation reaction (MOR) [120]. PtRu/NCNHs outperformed reference catalyst PtRu/Vulcan and commercial Pt supported on carbon catalysts against MOR and in tolerance to the carbonaceous intermediates. In an alternative simplified and straightforward approach, CNHs were grown directly onto conductive carbon microfibers by laser ablation [121]. Electrochemical studies of the Pt nanoparticles on CNHs catalyst demonstrated promising results for ORR and MOR. The same team did a more thorough study of the CNHs, focusing on the electroanalytical application. Intact CNHs were tested for the oxidation of ferrocyanide and exhibited higher peak current densities and lowest anodic peak-to-cathodic peak separation compared to other carbon samples [122]. Afterwards, CNHs were coated with Pt of different morphologies and studied as anodes for MOR and as cathodes for ORR with good electrocatalytic activity.

Some effective ways to address the problems that DMFCs face is to operate the fuel cell at high temperatures (above 120 °C) and provide the cell a mixture of methanol and water in the vapor phase. Vapor phase DMFCs show significant advantages, i.e., they show higher energy efficiency, enhanced mass transfer and anode electrode kinetics, they do not require methanol dilution (which makes the fuel tank smaller), and they show higher cathode tolerance to methanol crossover, while methanol crossover in the vapor phase decreases with temperature [123]. In this framework, CNHs were used as an electrocatalyst support in a vapor phase high temperature DMFC (HT-DMFC) and its performance was assessed [124]. The CNH-based electrocatalyst showed superior methanol electrochemical oxidation than CB and oxygen reduction reactions for voltages lower than 0.65V vs. RHE. The improved performance was attributed to higher water vapor adsorption and/or electrode morphology. Moreover, CNH-based electrodes presented improved performance and longer stability in a vapor phase HT-DMFC environment. Additionally, CNHs and carbon black supports were compared in membrane electrode assemblies (MEA) for high temperature PEMFCs [125]. Similar peak power densities were obtained in both cases operating at 160 °C. Nevertheless, Pt/CNHs hybrids showed a higher ohmic resistance compared to carbon black-based MEA, most likely attributed to the hydrophobic character of CNHs. Furthermore, the Pt/CNH anode exhibited lower charge transfer resistance, while the Pt/CNH cathode electrode presented similar cathode charge-transfer resistance than the corresponding CB electrode [126].

The electrocatalytic properties, characteristics, and performance of CNH-based materials towards MOR are summed up in Table 2.

### 2.3. Water Splitting—Hydrogen Evolution and Oxygen Evolution

Electrolysis is the process where water electrocatalytically dissociates to hydrogen, via the hydrogen evolution reaction (HER) and oxygen, via the oxygen evolution reaction (OER). Electrolysis is a keystone reaction toward a society that aims on becoming independent from fossil fuels. Hydrogen is considered to be a promising candidate for a secondary source of energy. Currently, 96% of hydrogen is produced by steam reforming from hydrocarbon fuels. An alternative way to address dependence on finite resources is the power-to-gas strategy, where intermittent energy resources are transferred and stored as hydrogen, mostly generated from water splitting. The latter reaction is a straightforward, inexpensive, and highly efficient approach to produce hydrogen and takes place in a unit called an electrolyzer. Water electrolyzers are expected to rank highly in the production of hydrogen in the coming years, for instance, in hydrogen-fueling stations. Water splitting can happen when applying electrical energy:

H_2_O → H_2_ + ½O_2_ (4)

In an electrolyzer, the above reaction is separated by an electrolyte (in liquid or solid form) into two half reactions. The HER occurs at the cathode:

2H^+^ + 2e^−^ → H_2_ (5)

while the other half reaction, OER, occurs at the anode:

2H_2_O → O_2_ + 4H^+^ + 4e^−^ (6)

The hydrogen evolution reaction can happen in three elementary steps in acidic media. Reaction, referred to as the Volmer step, is the first step of the reaction, where the reduction of a proton on an active site of the catalyst surface takes place. Then, the evolution of molecular H_2_ follows, either through a second proton/electron transfer (Heyrovsky step) or through recombination of two hydrogen atoms adsorbed on the electrode surface (Tafel step).
H_3_O^+^ + e^−^ → H_ads_ + H_2_O(Volmer adsorption)H_ads_ + H_3_O^+^ + e^−^ → H_2_ + H_2_O(Heyrovsky desorption)H_ads_ + H_ads_ → H_2_(Tafel desorption)

Tafel analysis is a very effective method to compare electrocatalytic activity and to elucidate the reaction mechanism of electrocatalysts. Analysis of the Tafel slope, i.e., the sensitivity of the electric current response to the applied potential can provide information associated with the rate determining steps of electrolysis. The experimentally observed Tafel slopes can be compared with the theoretically derived slopes assuming different rate-determining steps based on the microkinetic model. The kinetic parameters of the overall reaction, apparent Tafel slope, and reaction order, are determined by the rate-determining step and are subject to various criteria, such as the electrode material, surface crystal structure, electrolyte, overpotential, adsorbed species, and trace impurities [127]. Up to date, Pt and Pt-based materials are the most efficient electrocatalysts towards HER with excellent performance, since they exhibit almost zero onset potential and zero Gibbs free energy of hydrogen adsorption (ΔG_H_) and Tafel slope around 30 mV/dec. In general, favorable electrocatalysts exhibit low overpotential, low Tafel slope values, and should be able to withstand high current density as well as be resistant to acidic media in which normally HER takes place. Different non-noble metal electrocatalysts have undergone intensive investigation over the past years, such as alloys [128,129], transition metal compounds MX (where M is Mo, W, Ni, Co, etc., and X is S, Se, P, C, N, etc.) [130,131,132], and carbonaceous nanomaterials [133]. Meanwhile, carbon nanomaterials feature tunable molecular structures, interesting composition chemistry, superior conductivity, and negligible environmental impact, and thus, they have been massively used as electrocatalysts toward HER. Even so, these catalysts still have not been able to minimize overpotential like their transition metal-based counterparts. This is why carbon nanomaterials have mostly been used as electrocatalytic supports. As a matter of fact, electrochemical activation and/or acidic treatment has proven to help effectively carbon nanomaterials such as CNTs [134,135] and fullerenes [136], to report outstanding catalytic performances for HER, comparable to that of Pt/C [135]. Proposed activation mechanisms for the enhanced catalytic activity are the increase in the number of oxygen functionalities, which was considered as the active sites for HER [134], or the “adjacent Tafel” mechanism [135], but still, further investigation is required. In order to reach a better understanding of this mechanism, the activation of CNHs was investigated by using different counter electrodes during CV measurements [137]. In a three-electrode cell, current passes between the working electrode and the counter electrode. In theory, the counter electrode can be of any kind, since its electrochemical properties do not affect the behavior of the working one. However, when the Pt wire was used as the counter electrode, the electrochemical catalytic activity of CNHs towards HER was greatly improved and was even as good as the commercial Pt/C. This was attributed to the dissolution of the Pt electrode from the oxidation reaction (Pt^0^ to Pt^2+^), which is favored in long time tests under acidic medium. The dissolved Pt^2+^ transfers to the working electrode surface and is deposited on the electrode materials, which leads to increased catalytic performance. However, this was not observed when graphite rod was used as a counter electrode. The above findings demonstrate the importance of evaluating all parameters to avoid reaching misleading conclusions.

**Figure 4:**
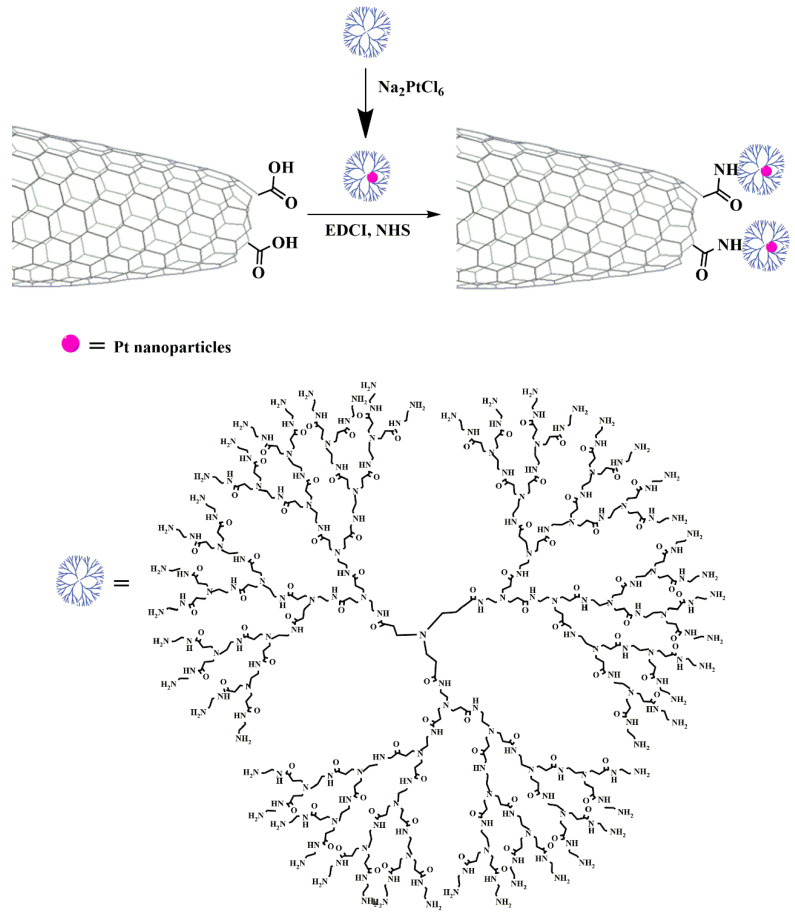
Schematic representation of the covalent bonding of PAMAM dendrimers encapsulating Pt nanoparticles on oxidized CNHs.

The turf of water splitting engaging CNHs as electrocatalysts remains quite unexplored, since only few works have been reported so far regarding this concept. In this regard, hybridization of nonprecious metals with CNHs by employing diverse designs and strategies is a highly appealing concept in order to enhance their performance [96,138]. Furthermore, there is no question that use of CNHs has been an effective way to minimize the loading of precious metals. In this route, Pt nanoparticles were immobilized on amine-bearing CNHs onto poly(amido amine) (PAMAM) dendrimers [139], as shown in Figure 4. The dendrimer was used as linkage for the stabilization of the Pt nanoparticles on the modified CNHs, but also as a stabilizer to prevent the agglomeration of the nanoparticles. The so-formed CNH-based hybrid material showed excellent HER activity, durability, and stability even with low loading (~1 wt%) of Pt, which was approximately 40 times lower than commercial 40 wt% Pt/C. The hybrid’s HER efficiency was comparable to 40 wt% Pt/C as well as to Pt/MoS2-based electrocatalysts consisting of 10 and 2.03 wt% Pt. In addition, the Volmer–Heyrovsky mechanism was found to be the rate-limiting step for as-prepared catalyst, whereas for Pt/C, the Volmer–Tafel mechanism characterizes the kinetics of the reaction.

The role of CNHs as electrocatalytic supports for MoS_2_ and polydopamine (PDA)/Pd nanoparticles was also studied and their performance towards HER was evaluated [140]. The presence of PDA ensured the uniform distribution of Pd nanoparticles on the surface of CNHs without aggregation, generating the PDA-Pd/CNHs hybrid material, whereas the thin coating of PDA reduced the inhibition for the reversible oxidation nature of Pd nanoparticles. In the same work, the MoS_2_ and CNH-based hybrid material, MoS_2_/CNHs, was acquired via the in situ preparation of MoS_2_ in the presence of CNHs by using a hydrothermal method [140]. In general, transition metal dichalcogenides (TMDs) are able to minimize overpotential for HER and lower Tafel slope values due to their innate catalytic activities [141]. Hence, they have been massively studied and employed in the development of electrocatalysts for HER with various architectures [142,143,144,145,146]. Moreover, carbon-based nanomaterials, like graphene and heteroatom-doped graphene, have been effectually combined with TMDs (MoS_2_, WS_2_, MoSe, etc.), further boosting their catalytic activity [67,147,148,149]. However, the MoS_2_/CNHs electrocatalyst showed poor performance for HER, attributed to the ineffective interactions between the two elements, since MoS_2_ sheets wrapped around CNHs, blocking the exposure of their defective sites. On the other hand, the contribution of CNHs to PDA-Pd triggered a superior hydrogen production than MoS_2_/CNHs. This was further screened from the lower Tafel slope value of the metal-based electrocatalyst (61 mV/dec) compared to MoS_2_/CNHs (86 mV/dec).

**Figure 5:**
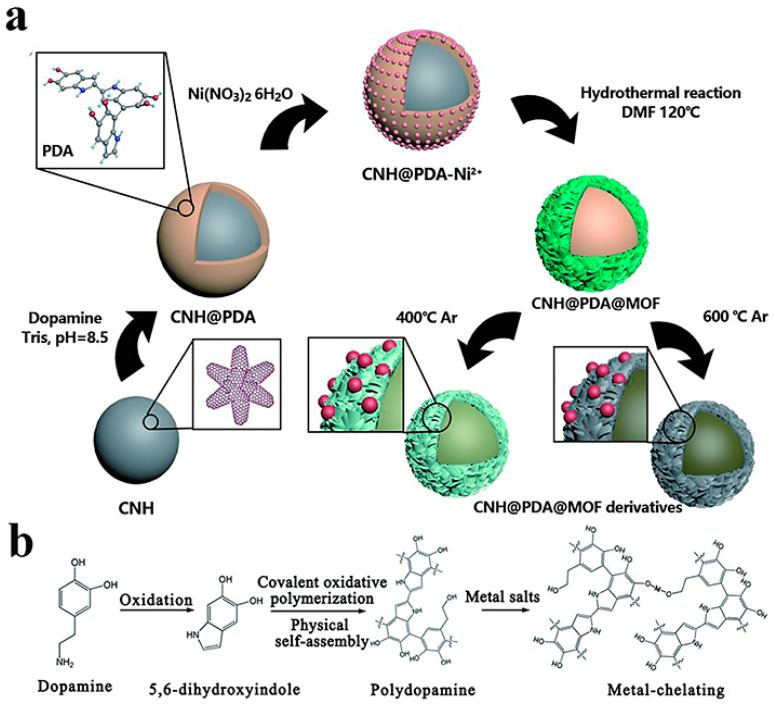
Schematic illustrations of the (**a**) synthetic route of N-CNH-Ni-MOF and its derivatives, and (**b**) polymerization of dopamine. Reproduced with permission from [150]. Copyright © American Chemical Society, 2020.

As far as the OER concerns, to the best of our knowledge, there is only a single report concerning CNHs. So far, the best OER activities have been reported for metal oxides such as ruthenium and iridium oxides. In seeking to take distance from these expensive and scarce materials, a double core-shell catalytic system combining N-doped CNHs, MOF, and Ni nanoparticles (N-CNH-Ni-MOF) was prepared by a simple hydrothermal reaction [150]. Incorporation of CNHs helps to overcome the poor stability of MOFs in high temperatures and polar solvents, while it also improves conductivity. On the other hand, the introduction of MOFs gives CNHs massive active sites on their surface, which could significantly promote their catalytic property. Hence, dopamine was self-polymerized to introduce multiple chemical functional groups on the CNHs surface and direct the nucleation and growth of MOFs, leading to well-defined core-shell structures. Furthermore, Ni nanoparticles were introduced to the catalyst by pyrolyzing the material, while treatment of dopamine in elevated temperatures led to N-doping of CNHs (Figure 5). The so-formed hybrid electrocatalyst, pyrolyzed at 400 °C, exhibited excellent stability and good electrocatalytic OER activity, outperforming the commercial noble-metal catalyst and the state-of-the-art Ni-based catalysts [151,152,153,154,155], but also the Ni-based graphene and CNT-based hybrids [156].

### 2.4. Other Electrocatalytic Reactions

**Figure 6:**
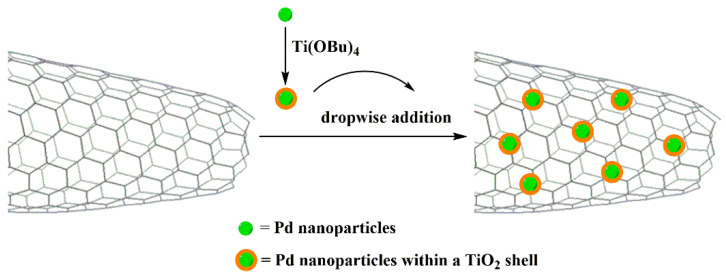
Schematic representation of the preparation of the Pd@TiO_2_/ox-CNHs heterostructure.

The CNHs have been also implemented in the electrochemical CO_2_ reduction reaction (CO_2_RR). The CO_2_ is a very stable molecule and requires high overpotential to start its reduction reaction [157]. At the cathode, CO_2_ is electrochemically reduced to chemicals or fuels, while the selectivity of CO_2_RR can be properly managed to produce formic acid as renewable fuel. In this frame, hybrid heterostructures were prepared by integrating Pd nanoparticles on oxidized CNHs stabilized by a TiO_2_ outer-shell (Pd@TiO_2_/ox-CNHs) [158], according to Figure 6. The Pd@TiO_2_/ox-CNHs hybrid material, when fabricated and employed as electrode, showed significant activity toward formate formation at low potential (~0.2 V vs. RHE), while hydrogen evolution was observed over time, as a product of the catalytic decomposition of formic acid accumulating in the system. In this system, CNHs acted as a scaffold, guaranteeing the electrical wiring of the Pd-active sites, and thus, boosting charge mobility. Although the bifunctional heterostructure showed high activity for generating formic acid, it also highly produced the CO intermediate. Moreover, the selectivity that the hybrid material showed toward formate formation was not as high compared to Sn or Bi metals [159,160].

The electrocatalytic properties, characteristics, and performance of CNH-based materials towards water splitting and CO_2_RR is summarized in Table 3.

## 3. Conclusions

There has been tremendous progress in recent years towards the exploration of innovating electrocatalysts for energy-related applications and particularly, in fuel cells. In this context, CNHs have their own state of play as novel electrocatalysts, since they possess intriguing properties deriving from their unconventional structure. The fact that their production is economical and can be done on a large scale renders them advantageous materials that can be utilized in energy-related applications, while in many cases, they are chosen over carbon nanotubes and even graphene. Although CNHs have been in the spotlight for quite some time, this interesting nanomaterial is kind of mistreated and has not reached its full potential yet, concerning electrocatalytic applications. CNHs have been employed as electrocatalysts, mostly as electrocatalytic supports, and have been tested primarily in oxygen reduction and methanol oxidation and less in the electrolysis of water. Interestingly, two major strategies have been used in order to increase electrocatalytic active sites and promote electrocatalytic activity, namely (i) doping of CNHs with heteroatoms, and (ii) hybridization with other catalysts. Doped and co-doped CNHs have shown promising results in ORR and MOR, outperforming, in some cases, the commercial Pt-based electrocatalysts. Meanwhile, immobilization of metallic nanoparticles onto CNHs can reduce the amount of noble metals, but also assists electrocatalytic performance, while a combination of CNHs and/or doped CNHs with alternative electrocatalysts, such as transition metal chalcogenides, has shown encouraging results for ORR. Therefore, CNHs bring benefits that can help in reaching superior performances in electrocatalytic reactions. Their unconventional structure, high purity and porosity, good electrical conductivity, and high surface area ensure an exceptional electrocatalyst for reactions that are of paramount importance in fuel cell technologies. Clearly, there is room for more in-depth investigations so that the unique properties of CNHs will be exploited in full.

## Figures and Tables

**Table 1:** Electrocatalytic properties, characteristics, and performance of CNH-based materials towards ORR.

Electrocatalyst	Reaction/Conditions	ORR Performance	Maximum Power Density	Ref.
Fe-CNH/CNT	ORR/ LSV in 0.1 M HClO_4_	Onset potential: 0.90 V vs. RHE Half-wave potential: 0.71 V vs. RHE Specific activity: 2.14 mA g/m^2^ at 0.7 V vs. RHE Electron transfer number: 3.89 Tafel slope: 77.4 mV decade^−1^ Durability: half-wave potential 0.67 V vs. RHE after 5000 cycles	200 mW/cm^2^	[56]
	ORR/ LSV in 0.5 M H_2_SO_4_	Onset potential: 0.90 V vs. RHE Half-wave potential: 0.75 V vs. RHE Tafel slope: 77.94 mV decade^−1^ Specific activity: 2.14 mA g/m^2^ at 0.7 V vs. RHE Electron transfer number: 3.95 Tafel slope: 77.94 mV decade^−1^ Durability: half-wave potential 0.73 V vs. RHE after 5000 cycles		
	ORR/ LSV in 0.1 M KOH	Onset potential: 0.1 V vs. RHE Half-wave potential: 0.85 V vs. RHE Specific activity: 17.39 mA g/m^2^ at 0.7 V vs. RHE Electron transfer number: 3.90 Tafel slope: 84.84 mV decade^−1^ Durability: half-wave potential 0.84 V vs. RHE after 5000 cycles		
N-CNHs	ORR/ LSV in 0.1 M KOH	Onset potential: −0.026 V vs. Hg/HgO Half-wave potential: −0.036 V vs. Hg/HgO * J_D_: −3.75 mA cm^−2^ at 1600 rpm Electron transfer number: 3.6 Tafel slope: 77.4 mV decade^−1^ Durability: 6% J_D_ loss after 1000 cycles	30 mW/cm^2^	[68]
N-doped CNHs	ORR/ LSV in 0.1 M KOH	Onset potential: 0.93 V vs. RHE Half-wave potential: ~0.84 V vs. RHE J_D_: ~−5 mA cm^−2^ at 1600 rpm Electron transfer number: 3.49	-	[69]
Sulfur-doped CNHs	ORR/ LSV in 0.1 M KOH	Onset potential: 0.6 V vs. RHE Half-wave potential: ~0.66 V vs. RHE J_D_: ~−2 mA cm^−2^ at 1600 rpm	-	[70]
N-B-CNH	ORR/ LSV in 0.1 M KOH	Onset potential: ~−0.21 V vs. Ag/AgCl Half-wave potential: ~−0.28V vs. Ag/AgCl J_D_: ~−2.8 mA cm^−2^ at 1600 rpm Durability: 4% current loss after 10,000s	-	[76]
N-P-CNH		Onset potential: ~−0.21 V vs. Ag/AgCl Half-wave potential: ~−0.28 V vs. Ag/AgCl J_D_: ~−2.5 mA cm^−2^ at 1600 rpm		
Fe-N-CNH	ORR/ LSV in 0.1 M KOH	Onset potential: 1.015 V vs. RHE Half-wave potential: 0.925 V vs. RHE J_D_: ~−5.6 mA cm^−2^ at 1600 rpm Electron transfer number: ~4 Durability: 4% loss of current density after 12h operation	250 mW/cm^2^/125 mW/cm^2^	[79]
Pd-N-B-CNHs	ORR/ LSV in 0.1 M KOH	Onset potential: −0.01 V vs. SCE Half-wave potential: −0.152 V vs. SCE J_D_: ~−5.8 mA cm^−2^ at 1600 rpm Electron transfer number: 4.2 Durability: 4.7% current loss after 9000 s	-	[86]
Pt/CNHs	-	-	2.5 mW/cm^2^	[92]
Pt/N-CNHs	ORR/ LSV in 0.1 M HClO_4_	Onset potential: ~1.0 V vs. RHE Half-wave potential: 0.904 V vs. RHE J_D_: ~−5.5 mA cm^−2^ at 1600 rpm ** J_k_: 2.15 mA cm^−2^ at 1600 rpm Mass activity: 0.305 A mg_Pt_^−1^ at 0.9 V vs. RHE Durability: half-wave potential 0.902 V vs. RHE after 15,000 cycles, J_k_: 2.09 mA cm^−2^ at 1600 rpm after 15,000 cycles	-	[95]
CoSe_2_/N-CNHs	ORR/ LSV in 0.1 M KOH	Onset potential: 0.9 V vs. RHE Half-wave potential: 0.81 V vs. RHE J_D_: ~−5.2 mA cm^−2^ at 1600 rpm J_k_: 8.08 mA cm^−2^ at 1600 rpm Mass activity: 13.8 A g^−1^ Electron transfer number: 4 Tafel slope: 52 mV decade^−1^ Durability: negligible losses for onset and half-wave potential after 2000 cycles	10.05 mW/cm/^2^	[102]
CNHs/MOF	ORR/ LSV in 0.1 M KOH	Onset potential: 0.97 V vs. RHE Half-wave potential: 0.87 V vs. RHE J_D_: ~−5.0 mA cm^−2^ at 1600 rpm Electron transfer number: ~3.7 Durability: 7% loss of current density after 12h operation	185 mW/cm^2^	[103]
N-Fe-doped CNH	ORR/ LSV in 0.1 M KOH	Onset potential: −0.09 V vs. Hg/HgO Half-wave potential: −0.026 V vs. Hg/HgO J_D_: ~−3.8 mA cm^−2^ at 1600 rpm Electron transfer number: 4 Tafel slope: 84 mV decade^−1^ Durability: Almost unchanged activity	35 mW/cm^2^	[107]
Co-g-C_3_N_4_/CNHs	ORR/ LSV in 0.1 M KOH	Onset potential: −0.066 V vs. Ag/AgCl Half-wave potential: −0.157 V vs. Ag/AgCl J_D_: ~−4.8 mA cm^−2^ at 1600 rpm Electron transfer number: 3.98 Durability: negligible activity loss after 12 h operation	-	[108]
g-N-CNHs	ORR-H_2_O_2_ production/CV-LSV in 0.1 M NaOH	Onset potential: 0.71 V vs. RHE Peak potential: 0.40 V vs. RHE Peak current density: −2.3 mA cm^−2^ Tafel slope: 71 mV decade^−1^ Electron transfer number: 3.1	-	[111]
	ORR-H_2_O_2_ production/CV-LSV in 0.1 M phosphate buffer solution	Onset potential: 0.53 V vs. RHE Peak potential: 0.59 V vs. RHE Peak current density: −1.79 mA cm^−2^ Tafel slope: 84 mV decade^−1^ Electron transfer number: 2.1		
	ORR-H_2_O_2_ production/CV-LSV in 0.1 M H_2_SO_4_	Onset potential: 0.40 V vs. RHE Peak potential: 0.18 V vs. RHE Peak current density: −3.6 mA cm^−2^ Tafel slope: 95 mV decade^−1^ Electron transfer number: 2.4		
Pt/CNHs	ORR/ CV in 0.5 M H_2_SO_4_	Peak potential: 0.33 V vs. Ag/AgCl	-	[121]
Pt/CNHs	ORR/ LSV in 0.5 M H_2_SO_4_	Onset potential: 0.77 V vs. Ag/AgCl Half-wave potential: 0.67 V vs. Ag/AgCl Current density: 34.54 mAcm^−2^ Mass activity: 50.06 mA mg_Pt_^−1^	-	[122]

* J_D_—diffusion limited current density; ** J_k_—kinetic current density.

**Table 2:** Electrocatalytic properties, characteristics, and performance of CNH-based materials towards MOR.

Electrocatalyst	Reaction/Conditions	MOR Performance	Ref.
Pt/CNHs	MOR/CV in 0.5 M H_2_SO_4_/1.0 M CH_3_OH	Oxidation peak: 0.7 V vs. SCE Mass activity: 350 mA mg^−1^ Durability: 90% mass activity loss after 1800 s	[118]
Pt/CNHs	MOR/CV in 1.0 M KOH/1.0 M CH_3_OH	Oxidation peak: −0.22 V vs. Ag/AgCl Mass activity: 0.49 mA μg^−1^ Durability: 13% peak current loss after 100 cycles	[119]
	Formic acid oxidation reaction/CV in 0.5 M H_2_SO_4_/0.5 M HCOOH	Oxidation peak: 0.35 V vs. Ag/AgCl Mass activity: 81 mA mg^−1^ Durability: 60% mass activity loss after 3600 s	
PtRu/N-CNHs	MOR/CV in 0.5 M H_2_SO_4_/1 M CH_3_OH	Oxidation peak: 0.7 V vs. RHE Mass activity: 850 mA mg^−1^	[120]
Pt/CNHs	MOR/CV in 0.5 M H_2_SO_4_/1 M CH_3_OH	Oxidation peak: 0.7 V vs. Ag/AgCl	[121]
Pt/CNHs	MOR/CV in 0.5 M H_2_SO_4_/1 M CH_3_OH	Oxidation peak: 0.69 V vs. Ag/AgCl Current density: 127 mA cm^−2^ Mass activity: 184 mA mg_Pt_^−1^	[122]

**Table 3:** Electrocatalytic properties, characteristics, and performance of CNH-based materials towards water splitting and CO_2_RR.

Electrocatalyst	Reaction/Conditions	Reaction Performance	Ref.
CNHs	HER/LSV in 0.5 M H_2_SO_4_	Onset potential: ~0.53 V vs. RHE	[137]
CNH/PAMAM-Pt	HER/LSV in 0.5 M H_2_SO_4_	Onset potential: −0.016 V vs. RHE Current density at 50 mV: 2.5 mA cm^−2^ Tafel slope: 42 mV decade^−1^ Durability: Same HER activity after 500 cycles	[139]
PDA-Pd/CNHs	HER/LSV in 0.5 M H_2_SO_4_	Onset potential: ~−0.01 V vs. RHE Potential at 10 mA cm^2^: ~−0.12 vs. RHE Tafel slope: 61 mV decade^−1^	[140]
MoS_2_/CNHs		Onset potential: ~−0.22 V vs. RHE Potential at 10 mA cm^2^: −0.246 V vs. RHE vs. RHE Tafel slope: 86 mV decade^−1^	
N-CNH-Ni-MOF	OER/LSV in 1.0 M KOH	Onset potential: 0.07 V vs. RHE Potential at 10 mA cm^2^: 0.320 V vs. RHE Tafel slope: 85.3 mV decade^−1^ Durability: Same electrocatalytic activity after 15 h	[150]
Pd@TiO_2_/ox-CNHs	CO_2_RR/CV in 0.1 M phosphate buffer solution	Onset potential: −0.05 V vs. RHE Tafel slope: 149 mV decade^−1^ Durability: Same electrocatalytic activity after 48 h of electrolysis	[158]
